# Healthcare Communication Experiences of Hispanic Caregivers of Childhood Cancer Survivors

**DOI:** 10.3390/healthcare12131307

**Published:** 2024-06-30

**Authors:** Carol Y. Ochoa-Dominguez, Matthew P. Banegas, Kimberly A. Miller, Carlos Orellana Garcia, Daniel Sabater-Minarim, Randall Y. Chan

**Affiliations:** 1 Department of Radiation Medicine and Applied Sciences, University of California San Diego, La Jolla, CA 92093, USA; 2 Center for Health Equity Education and Research, University of California San Diego, La Jolla, CA 92093, USA; 3 Department of Population and Public Health Sciences, Keck School of Medicine, University of Southern California, Los Angeles, CA 90007, USA; 4 Department of Dermatology, Keck School of Medicine, University of Southern California, Los Angeles, CA 90007, USA; 5 Department of Biological Sciences, University of California San Diego, La Jolla, CA 92093, USA; 6 Department of Pediatrics, Los Angeles General Medical Center, Los Angeles, CA 90033, USA; 7 Department of Pediatrics, Keck School of Medicine, University of Southern California, Los Angeles, CA 90007, USA

**Keywords:** caregivers, childhood cancer survivors, communication

## Abstract

Background: Interpersonal communication is a crucial component of the cancer experience that can contribute to managing cancer care and improving cancer survivors’ and caregivers’ quality of life. Cultural and contextual factors may impact Hispanic childhood cancer survivor (CCS) and parent caregiver relationships and communication. This study sought to describe the healthcare communication experiences of Hispanic parents with CCS, families, and medical providers. Methods: We conducted 15 semi-structured interviews with Hispanic caregivers from a safety-net hospital in Los Angeles County. Interviews were conducted in English and Spanish, audio-recorded and professionally transcribed, and analyzed using a thematic approach. Results: Caregivers shared the importance and impact of medical communication when exploring the “first big talk” of the diagnosis, uncertainty about treatment, navigating multiple providers, therapeutic communication (i.e., providing emotional reassurance), and current and lingering effects of cancer. All caregivers shared “good communication” experiences, while others shared various barriers to communication, including a lack of understanding of the cancer diagnosis and caregiver experience, psychological challenges impacting communication, cultural and language differences, physical factors that limit communication, and young age of child impacting communication with caregivers. Conclusions: Our findings suggest that a strong interpersonal communication skill set for clinicians can contribute to managing cancer care and improving caregivers’ psychological adjustment.

## 1. Introduction

Communication plays an important role in the experiences and outcomes of patients with cancer, during and after treatment. Previous research suggests that having good communication with trust and expressing concerns between the patient–provider, patient–caregiver, and caregiver–provider results in comfortably sharing their needs, which leads to quality-of-life improvements [1,2,3]. Including patients and caregivers in the health-related decision-making conversation may also help patients feel more empowered, improve their health literacy skills, and better transition to care after cancer treatment [3]. For example, by communicating cultural factors that impact the lives of underrepresented patients, they may be more likely to feel safe to express concerns or barriers impacting their care [1,2,4].

It has been shown that caregivers experience emotional strain after diagnosis, adversely impacting the comprehension of provided education; they feel overwhelmed and have compared the cancer learning curve to learning a new language [5]. While caregivers desire comprehensive information, they are often too overwhelmed at diagnosis to receive it effectively; thus, the need for ongoing information provision persists for years after diagnosis and the waning influx of information after the initial diagnostic period can lead to dissatisfaction [6]. Even after the end of curative therapy, caregivers have expressed the continuing need for good information and communication. Fear of relapse and ambiguous symptoms have been reported to be primary concerns, although caregivers also fear seemingly inevitable loss of connection with their primary oncologist [7]. Interestingly, despite these perceived deficits, caregivers consistently report receiving quantities of information beyond their capacity to process [8].

Nonetheless, communication barriers may arise that impact the needs and quality of life of the patient and caregivers, particularly patients who identify as Hispanic or Latino [4,9,10]. Previous studies have mentioned that for Hispanic/Latino caregivers of childhood cancer survivors (CCSs), cultural differences (e.g., language, religious practices, health beliefs), physical factors, and lack of understanding are communication barriers for many families as they are seeking quality cancer care for their children [11,12]. Language barriers create hesitation and psychological distress within non-native caregivers when expressing their demands or concerns with their providers to obtain culturally appropriate resources and healthcare for a better quality of life [11,12]. Lack of understanding is another communication barrier that can impact interaction within families, between families, and interaction with clinicians; these communication barriers can be due to cultural differences, language, or health literacy, which create tension in interaction and negatively impact patients’ preparedness to manage their care [4,9,10,11,12]. Physical barriers to communication will often impact families’ receipt of care and may include transportation, insurance, legal status/documentation, and housing concerns [4]. Psychological barriers can impact Hispanic/Latino patients and family members by creating a sense of fear in communicating about cancer or the experiences within treatment due to the psychological distress and trauma it represents [13,14,15,16]. These various barriers make it difficult for the transition to care and for young adult CCS to have stronger health literacy and self-efficacy [4,17]. Finally, the age of the CCS can be a communication barrier, since the patients themselves are too young to express their needs, experiences, and understanding, while the parents also are not fully able to explain cancer diagnosis or treatment [9].

The need for effective communication is crucial during the trajectory of cancer care and survivorship. Communication may be carried out between the healthcare team (e.g., physicians, nurses, social workers), CCSs, spouses, and family members [4,9,10]. These interactions may include discussing cancer diagnosis, the uncertainty of treatment, and the current/lingering effects of cancer. Other communication topics may consist of future goals, returning to normalcy, and social needs. To obtain different support through psychological distress or networks for better resources and recommendations, Hispanic/Latino caregivers communicate with their friends and other CCS caregivers; through these interactions, they can gather resources that match their quality of life, share any fears or concerns, and self-advocate for their family’s well-being [10,14]. In addition, many caregivers will get closer to their religion and communicate with higher beings to obtain comfort and support during these stressful times [13]. This paper aims to describe the communication experiences of Hispanic/Latino caregivers with CCSs, families, and medical providers, as discussed through qualitative interviews. Furthermore, we cover important communication concepts and barriers discussed by Hispanic/Latino caregivers.

## 2. Methods

### 2.1. Research Design

We conducted 15 in-depth, semi-structured interviews focusing on Hispanic/Latino parents or caregivers of childhood cancer survivors. Participants were chosen through purposive sampling from the Los Angeles General Medical Center (previously named LAC + USC) in Los Angeles, which was previously discussed in greater detail [18]. The selection criteria included (1) being a primary caregiver to a child who had completed cancer treatment, (2) self-identification as Hispanic/Latino by the caregiver or child, and (3) proficiency in either English or Spanish. Recruitment methods included direct in-person approaches during clinic visits and phone calls via the child’s medical oncologist (RYC). Interested caregivers were subsequently contacted by the principal investigator (PI: CYOD) for a comprehensive study overview, informed consent process, and interview scheduling. From July to September 2020, these interviews were performed in the participant’s preferred language and audio-recorded for accuracy. Each interview lasted 45 to 60 min and concluded with a demographic survey; all participants were compensated with a USD 25 gift card. Interviews were conducted until thematic saturation was reached. The entire procedure received approval from the USC Institutional Review Board (IRB).

### 2.2. Interview Guide

The interviews were structured around a guide developed from an extensive review of the existing literature, aiming to explore four vital facets of the caregiving experience, which included (1) barriers and facilitators to caregiving, (2) social support, (3) psychosocial adjustment, and (4) communication. The following relevant questions focused on communication experiences: (1) How openly is your child’s cancer diagnosis and treatment discussed between you and your child? What about in your household? And with doctors? (2) Can you tell me about what type of cancer-related things you have talked about with your child, family, or doctors? (3) Was there ever a time during your child’s cancer treatment that you felt that you were unable to understand your child’s medical provider? (4) Was there ever a time during your child’s cancer treatment that you felt that you were unable to discuss any health issues about your child? The guiding questions were open-ended, based on a literature review, and the entire interview guide is available as Supplementary Files S1 and S2.

### 2.3. Analytical Strategy

We employed reflexive thematic analysis, as described by Braun and Clarke [19], with elements of grounded theory methodology to ensure the capture of meaningful themes [20]. The research team utilized Dedoose software version 9 to analyze anonymized interview transcripts. DSM and COG, both trained in qualitative data analysis, read the transcripts to grasp the caregivers’ narratives and patterns in the data. Coding was conducted inductively and independently, focusing on explicit content to identify key codes and themes.

Subsequently, DSM and COG collaborated with the principal investigator (CYOD) to discuss and refine these initial codes. This collaborative phase also involved seven additional meetings with team members MPB, KAM, and RYC to finalize the codebook on caregiver communication experiences. This iterative process ensured the development of accurate themes and subthemes, from which relevant quotes were selected for this study. Finally, the established codebook guided a comprehensive re-examination of all interviews, ensuring a thorough and nuanced understanding of the data.

## 3. Results

### 3.1. Participant Characteristics

The study participants consisted primarily of mothers (93%) who were Spanish-speaking (67%), ranging in age from 23 to 58 years. These participants were mostly caring for children who had been diagnosed with leukemia and were at least two years post-treatment. Common demographic characteristics included having less than a high school education, an annual household income under USD 40,000, and reliance on public health insurance. Demographics are summarized in Table 1.

### 3.2. Major Findings

**Figure 1:**
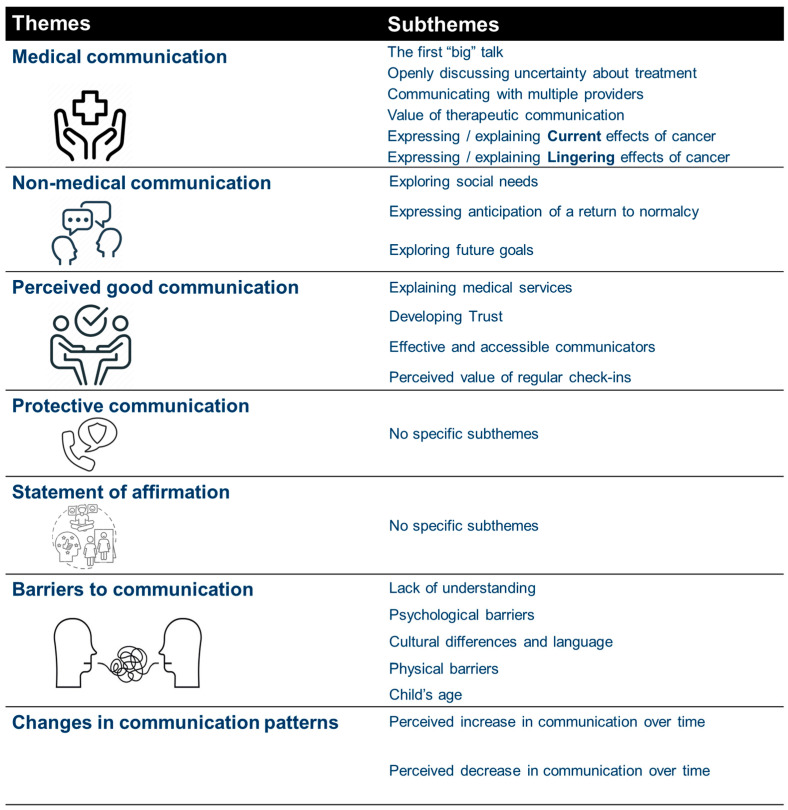
Summary of Themes and Subthemes.

Seven themes were identified related to communication, including (1) medical information, (2) non-medical discussions, (3) perception of good communications, (4) protective communications, (5) statements of affirmation, (6) communication barriers, and (7) changes in communication (see Figure 1).

#### 3.2.1. Medical Communication

All caregivers shared the importance and impact of medical communication. This encompassed any form of communication (e.g., face-to-face conversation, telephone, email, or a medical record) related to medical care needs, treatment options, or CCS health.

The majority of the caregivers vividly recalled the “*first big talk*”, the initial conversation in which they found out about their child’s cancer diagnosis and treatment options. Additionally, most caregivers remembered having to decide who would tell their child the news (e.g., themselves or the doctors) and whether to use the word cancer or to utilize vague terminology. For example, caregiver #9 shared: “[The doctors] told [my child]… with cartoons…with books…and…a stuffed animal. [Prior to placing] a central line to give her the medication, they [put a fake catheter on] her, and… the stuffed animal…to explain to her that through that tube she had… they were going to give the medicine”. Caregiver #13 shared that “…they [spoke to my daughter] in English, and then I told her that I wanted an interpreter so they could explain to me in Spanish, [so] they called an interpreter”. While caregiver #11 shared that “the doctor…gave the diagnosis to me and his dad at the hospital. And then he said to me: ‘do you want me to talk to [your son] and tell him what is happening with him and what his diagnosis is?—Or—Do you want to do it?’ And I said, ‘No, you can do it.’”.

About half of the caregivers discussed their *uncertainty about the treatment’s* impact on their child’s health. Specifically, caregivers described how it was difficult to have conversations with doctors and their families when the treatment outcome was uncertain. For example, caregiver #6 shared that “…seeing [her son] getting ill with the chemotherapy… you [don’t] want to see your child like that, it’s not making them feel good. [Everything] flies over [your] head, but [you] know they have to do what they have to do”. Caregiver #2, shared that while her child was initially diagnosed and treated in Mexico, she was told that chemotherapy was not working, that she would need a bone marrow transplant and the wait time could be over a year. Therefore, that caregiver told her doctor, “I’m not going to wait and see [if] she’s dying”, and then she decided to leave for the United States the next day.

Half of the caregivers discussed the use of *therapeutic communication* (i.e., providing emotional or mental reassurance) encompassing a range of verbal or non-verbal exchanges that a healthcare provider or parent might use to support the CCS or parent emotionally and mentally. Modalities described included validation of feelings, reassurance, the sharing of perspective, and reinforcing a positive outlook. Several caregivers noted that therapeutic communication was often their only tool to provide support. For example, caregiver #3 shared, “…[I] had to become a strong parent and tell him everything is going to be okay…it’s going to go away, all this is going to pass. You are strong. You are a warrior”. Caregiver #14 discussed that her son worried about cancer recurrence, and she would tell him “to be positive. If he did it once, he could do it again. [He] just had to be a strong little boy”. Additionally, caregivers described helping each other cope. Parents described seeing new families and wanting to help, as “[their] world collapses and [they] think that everything is already over. In particular, caregiver #1 recounted that “I began to talk to the mothers of the new patients who were arriving. [I would] encourage them and tell them to have faith. [I would tell them], that everything was going to be okay, [and] that my children had gone through the same thing”.

The majority of caregivers discussed the *current and lingering effects of cancer* with the healthcare team and CCS. *Current effects* were commonly reported, as 12 out of the 15 caregivers mentioned that their child experienced behavioral symptoms (e.g., mood swings, lack of attention span), mental health deterioration (e.g., depression, anxiety), and physical effects (e.g., inability to walk, alopecia, anorexia, unintentional weight loss, physical weakness) from diagnosis through treatment. Caregiver #14 recalled describing her son’s experience with the healthcare team; she relayed that “he stopped walking completely, [I have to] carry him…to move him around”, and afterward, “it was like he was a baby learning how to walk again”. In contrast, caregiver #4 reported “not [talking] much [about] the side effects [with my son]. When his hair was falling off, [I] just decided to shave his head. [I] just let him know that at the moment because he was sick, [I] just needed to cut his hair. Once [therapy] were done, his hair was going to grow back, and he [would] be fine”. Multiple caregivers reported their child experienced mood swings (often attributed to medications such as glucocorticoids); while the healthcare team talked to them about it, one caregiver expressed that she was still caught by surprise as she “didn’t know [how] extreme it would be”.

Along the same lines, *lingering effects* were also common; 10 out of the 15 caregivers shared discussing these with the healthcare team, their child, and others. *Lingering effects* consisted of any mention of post-treatment/late effects of cancer diagnosis (e.g., long-term effects, school performance, cancer recurrence, fertility). For example, caregiver #8 recalled her daughter asking her, “Do you think if one day I get married, [that] I will [still be able] to have children because of the chemotherapy?”. Similarly, caregiver #6 shared how her son asked her “if [I] wanted to be a grandma one day, and [I] told him that because of his chemotherapy and what doctors have told [me]… there is a slight chance that he might be sterile”. A few caregivers discussed how the treatment their child received was “very strong, and sometimes it affects their ability to learn”, so they found themselves communicating with both the healthcare team and the school staff to advocate for their child. Caregivers described fighting with the school districts to get accommodations such as an individualized education plan (known as an IEP). Some caregivers described their children as “being held back a year” or “being diagnosed with Attention Deficit Hyperactivity Disorder”. Multiple caregivers emphasized teaching their children to care for themselves, to learn to constantly be aware of their bodies, and how to seek emergency help in case of acute illness. For example, caregiver #9 recalled telling her daughter that “[you] have to know [your] body. If [you] have pain [or] a fever or something like that, [you have to] run to the hospital or call the doctor…and [you have] to tell us”.

A few caregivers shared that *navigating communication among multiple providers *could be complex, particularly in situations requiring emergency care. Caregivers spoke of the anxiety of meeting new doctors who were unfamiliar with the medical histories of their children when they were acutely ill—sometimes near death. In the case of caregiver #15, she reported that it was “helpful” when she would take her child to an emergency department (ED) where they had a record of her child’s medical condition, and they knew the oncologist. At the ED, caregivers reported doctors calling the oncologist “as soon as they had the results…to see what she wanted to do, like if she wanted to keep her, leave her, take her, send her home, and things like that”. However, caregivers reported a very different experience if they sought care at a medical facility that did not have access to the child’s medical records. Beyond a simple unfamiliarity with the child’s history, caregivers report feeling “ignored”, and one caregiver described how she did not like how the staff treated her child.

#### 3.2.2. Non-Medical Communication

Caregivers described frequently communicating about various non-medical aspects of life impacted by the cancer diagnosis. According to caregivers, this type of communication included *social needs, returning to normalcy, and future goals*.

Caregivers shared that they discussed their *social needs* with the healthcare team, family, and other individuals. Among the caregivers who experienced social needs, they described difficulties with housing, transportation, meeting financial obligations, and challenges with employment. Frequently, the cancer diagnosis and treatment exacerbated pre-existing socioeconomic difficulties. The following quotes reflect their experiences:

“…I was [at the hospital] all the time. I stopped working… And the sad part is [that] when I tried applying for [assistance], so they could help me… I didn’t qualify for [any] type of help… The [hospital staff] were the ones that [told me about assistance programs] and helped me [to] get [the] application… [I went] to the Social Security office [to] ask [about] that program, but that’s where they told me that [my child] didn’t qualify because he was not connected to a machine”.

(Caregiver #14)

“…I [have] had to deal with a lot; [I was] kicked out from where I used to live [and had to find] a place to live while having my son…in [the] hospital. It was very hard to find someone to want to help [me], or [to] help [me] find resources for myself or my son or my daughter…[Furthermore], I was going hungry in the hospital while they were taking care of [my child]… Hospitals don’t take into consideration that… people like me that have little to nothing…before that happens, we don’t [know] whether we eat that day or not… They would give you food for the patient, [but] there was no really help or resources for… being able to get food [for yourself while], being in the hospital”.

(Caregiver #6)

Caregivers also discussed communicating about *returning to normalcy* and highlighted the transition back to their regular day-to-day activities, which included the CCS returning to school and family-oriented routines. One caregiver shared a conversation with the father of her son and family about not spoiling him: “[just] because he was sick…everybody wanted to buy him everything. [How would I get] him back to…not just getting what you want when you want it…? You know, [back to] earning stuff and doing things because you’re being good” (Caregiver #4). Another caregiver described struggling with disciplining her son; she shared, “I had to tell him: ‘you were sick [but now] you’re not in the hospital anymore. [It is] one thing [when] you are sick, and another [when] you don’t even want to get up or wash your plate. There are things that you have to…know how to do…And at the very least, ‘you have to wash your dishes, you have to keep your room tidy, there are things you have to keep doing’” (Caregiver #3). Some caregivers shared having regular communication with the school staff about their child’s health and when they would be able to return to school and request accommodations. Other caregivers mentioned talking with their children about reintegrating back into their social lives. For example, caregiver #3 shared, “I told my son, ‘you have to go out, go [see] your friends,’ He tells me, ‘I do go out… with my friends.’ But I look at him…and…he’s gotten used to being locked up [and by himself]”.

A few caregivers added that they communicated about *future goals* with their children. These conversations included discussions about school, profession, and a desire to give back to their community. Caregiver #3 shared that “My son is thinking about [his] options…his brother had suggested if he doesn’t think he is going to do anything here…to also look for something in the Army, or in the Navy, or in the Air Force”. She also suggested that he look “on the internet for career programs that are a few months [in duration]”. Similarly, caregiver #6 explained, “We always talk about all his options if he’s good at something, you know, [he should] just go ahead and pursue it [especially] if [he is] happy, and like[s] [the job] …And I always tell him [that] if he can beat [cancer], [then] he can do whatever he wants. That there’s nothing stopping him from doing what he wants”. Lastly, caregiver #1 recalled that her child told her that she wanted to give back to other cancer patients: “I want to buy a good modern Nintendo and take it to the hospital where I was because when I was hospitalized, they had some that were very old, that’s why I didn’t play”.

#### 3.2.3. Perceived Good Communication

All caregivers shared “good communication” experiences; typically, that meant they were able to easily understand the information presented to them and/or they were able to obtain desired information from others (e.g., members of the healthcare team). Perceived good communication included the following: *healthcare team explaining medical services*, *trusting the healthcare team, effective and accessible formats of communication, and regular check-ins*.

The majority of caregivers shared that their child’s healthcare team did a good job of *explaining medical services* to them. This involved receiving information that was delivered clearly, in a way that they saw as appropriate. Caregivers desired digestible information with sufficient detail, especially regarding various topics (e.g., treatment, symptoms, preventive measures, and supportive services). Caregiver #11 described this encounter: “The oncologist explained to us what the steps were and how the [chemotherapy cycles] were going to go…he told us that when he goes into the second [month], his hair will start to fall out. And he explained everything to us very well. He also gave us options. He said: ‘I have to give you the option… [of] stay[ing] in this hospital or [going] to the children’s hospital.’”. Similarly, caregiver #10 shared that when the doctor explained about the treatments, he would also tell her “this treatment sometimes affects another area of the body. Everything has its risk”, and she appreciated his honesty. Caregiver #4 described, “[the] nurses were very helpful. And every time, you know, they explained something to me (e.g., how to read his temperature, how to give him his medicine, etc.), [they were] teaching me… [so that] when I went home, it would be easier for me, which I think was really helpful”. Lastly, caregiver #13 explained, “‘When they gave [my daughter] a prescription and instructions on how to take [the] medicine’… I would ask for an interpreter because ‘all that [needs] to be explained well and [I need] to understand well’”.

Additionally, most caregivers discussed how *having a relationship with, trusting, and feeling comfortable with the healthcare team* helped facilitate bidirectional communication. Caregivers indicated that the healthcare team built trust by providing credible information, showing compassion during conversations and in communication, and creating a safe environment where caregivers felt comfortable asking questions and contributing information. Examples of how caregivers described trusting the healthcare team include the following: “I [was able] to open up to the doctor” (caregiver #5); “I felt safe with that doctor; …she knew what she was doing. She wasn’t going to do something just because it’s, you know, something to try. She made me feel secure.” (caregiver #6).

Caregivers’ perception of good communication was bolstered when they experienced *effective and accessible* interactions. Caregivers expressed appreciation for the reception of multiple formats of communication, as they wanted information that was clear, direct, and easy to understand. In one example, as described by caregiver #9, whose daughter was very young when she received her cancer diagnosis, “the [healthcare team] used cartoons, books, and a stuffed animal to explain to her…[how] they were going to give her medicine [to treat her]”. Other caregivers reported that the availability of paper forms in English or Spanish for instructions and medical education on prognosis was very helpful for them, as they could review information as needed in the language that they were most comfortable reading. Additionally, Spanish-speaking caregivers mentioned that while many members of the healthcare team were bilingual, the availability of easily accessible professional translators felt both necessary and helpful.

Lastly, caregivers mentioned that *regular check-ins* contributed to their perception of good communication. These check-ins consisted of proactive and recurring conversations in which one person verbally or non-verbally communicated with the other to see how they were doing and if they had any unmet needs or health issues. For example, caregiver #14 shared that the healthcare team would call them to see how their child was feeling after a chemotherapy session. Caregiver #15 mentioned that the social worker would always check on them and would ask if the family needed any form of support or help. Other caregivers also shared that they would regularly check in with their child; they would ask about how the child was feeling because they knew that it was crucial to always monitor their child’s health during and after therapy.

#### 3.2.4. Protective Communication

In some cases, caregivers described experiencing unwarranted and unwelcome judgment regarding their child’s health and their decision-making, and they felt the need to verbally protect their decisions and viewpoints. Caregivers indicated participating in this *protective communication* with their family members, close friends, and, at times, with the healthcare team. In the case of family members and friends, caregivers felt judgment regarding differences in medical viewpoints and, more often, lifestyle choices. Some caregivers reported refusing visits from family and friends because of their child’s delicate health status. This occurred both before and after the COVID-19 restrictions began. Others engaged in protective communication with the healthcare team when they experienced disagreements about their child’s diagnosis, treatment, or health. Caregiver #7 stated, “One time, I got so angry, and [I] called the doctor’s attention. I said: ‘If you don’t have patience with children like that, let someone else who has patience [treat them].’”. In another case, caregiver #2 explained how she engaged in protective communication when she was trying to determine what was wrong with her daughter. She described taking her daughter to various doctors in Mexico, and they kept dismissing her symptoms, but she knew something was wrong and kept asking the doctors to evaluate her daughter until she was finally able to get a diagnosis in Mexico.

#### 3.2.5. Statement of Affirmations

Many of the caregivers commented on how they often utilized *statements of affirmation*, with themselves and their children. Caregivers found these statements to be helpful to shift away from a negative mindset, which they felt could help them overcome difficult situations. Common words of encouragement reported by caregivers included variations on “think positive and everything will be fine”, or “[let us] be positive that he will be cured”. Caregiver #3 shared, “I tried to make him feel that he was the strongest of all and that he could do it. I would tell him, ‘Everything is going to be fine…all of this is going to pass. You are strong. You are a warrior.’”. Caregivers also described instances where they reflected on their personal journey with their child’s cancer and their resiliency. For example, caregiver #8 shared that after going through the cancer journey with her daughter, when she would start to get anxious and nervous, she would tell herself, “I’m fine. …I know that I’m going to be fine, and I know that everything is going to be fine”.

#### 3.2.6. Barriers to Communication

Over half of the caregivers shared various barriers to communication, including a *lack of understanding of the cancer diagnosis and caregiver experience, psychological challenges impacting communication, cultural and language differences, physical factors that limit communication, and the young age of a child impacting communication with caregivers*.

Generally, caregivers mentioned experiencing barriers to communication with family and friends because of a *lack of understanding* about the cancer diagnosis and caregiver experience, due to an overall lack of medical knowledge and/or discordant health beliefs. For example, caregiver #8 recalled how her daughter felt rejected and avoided social contact as a result. This caregiver shared that her daughter told her, “I don’t want to go out… because I don’t want them to ask what I have…or tell me that if they touch me if something is going to happen to them…—Is cancer contagious?”. And I told her: “Cancer is not contagious, daughter. Only God knows why it happens, but it is not contagious”. Similarly, other caregivers expressed feeling rejected when friends and family would cease contact, which they believed was due to misunderstanding regarding the cause of cancer or due to fearing the delicacy of their child’s health.

Caregivers frequently mentioned that *psychological challenges* often hinder their communication with others, including the CCS, their spouse, family, friends, co-workers, and the healthcare team. Emotional and mental issues resulted in a sense of absent-mindedness, and thus, caregivers report having experiences of poor retention of information that was being communicated. Caregivers also reported a fear of expressing their feelings or thoughts to others, which directly impacted the quality of their communication. Most caregivers reported an avoidance of engaging others in conversation due to an adverse emotional state (e.g., fear, pain, anxiety, stress). Additionally, caregivers mentioned how perturbations in their child’s psychological health, including depression, anxiety, and behavioral issues, made it hard to communicate with their children or discipline them. Other times, caregivers shared explicitly being unable to process information shared by the healthcare team due to the emotional impact of their child’s cancer diagnosis.

A few caregivers stated that *cultural and language differences* created conflicts and/or deterred communication. Some parents recalled their child being told directly about their cancer diagnosis because the parents did not speak English and there was no interpreter available. Even when there was an interpreter available, caregiver #4 shared, “I think [because of] the language barrier, it’s … frightening [not] to know … if you understood right or if you didn’t”. Other caregivers described how cultural differences in health beliefs and religiosity manifested in friends, family, and other social contacts making insensitive comments. Some caregivers described such comments as directly exerting pressure on them to make specific decisions regarding their child’s health and therapy against medical advice, for example, stating that the caregiver should stop their child’s treatment or refuse blood transfusions, etc.

*Physical factors *also caused communication barriers for some caregivers. These consisted of long distances between family members, COVID-19 guideline restrictions that restricted caregivers from easily leaving and returning to the hospital or even restricting visitors to a single caregiver for an entire hospitalization, and also a lack of common space for caregivers to congregate at the hospital. Single parents faced very significant barriers that led to drastic changes in communication patterns. Caregiver #4 shared that she had her other three children move to their grandparent’s house after her child’s diagnosis so that she could focus on her child with active cancer. Therefore, the entire family had to “adjust to communicating by phone because [I] couldn’t [be] there physically [for them]”. Caregiver #6 shared that her communication and her relationship with her husband were hindered by his incarceration, which proved to be a significant barrier to his ability to provide support and understanding.

Over half of the caregivers expressed not being able to fully explain the cancer diagnosis and treatment to the CCS and their other children as they were too young to understand; thus, the *child’s age* acted as a barrier to caregivers’ cancer care communication. For example, caregiver #4 shared that “at the [time of diagnosis and treatment] he was four [years old], so it’s…harder for him to understand exactly… what was going on… I didn’t…really explain into detail just because I [felt it was inappropriate]”. Similarly, caregiver #5 explained that his son was two and a half years old when he was diagnosed, so “every time we were getting his medication or his chemo, I [told him that] he’s getting medicine for him to feel better”. Additionally, caregiver #15 mentioned having to convince her child every time that the treatment was good for her health. She recalled, “she was so little…she was just three…so I didn’t really explain to her…. [But] I’m like, you have to do it”.

#### 3.2.7. Changes in Communication Patterns

Some caregivers revealed that they experience a change in the frequency with which they communicate with cancer survivors, their partners, God, and co-workers. Specifically, three caregivers shared that *communication increased* after their child’s cancer diagnosis. Topics discussed included the child’s health, healthcare procedures and future care, self-motivation, “[gratitude] to God”, and routine check-ins. Several caregivers also shared that there was a *decrease in communication* after their child’s cancer diagnosis with family members and friends, often perceived as family and friends “distancing themselves”. Caregiver #6 explained that she believed this was due to “a lack of understanding and empathy”. Caregiver #1 shared her discussion with her family about her feelings of abandonment. She shared that the family response was the following: “[We have not made contact] because we couldn’t come over, because of [the child’s] condition. You see that his health was very delicate; we couldn’t go and bring him any bacteria”. And the caregiver responded: “Ok, I understand, but a call, a call, that wouldn’t affect the child”. Caregivers also described decreases in communication between the caregiver and the cancer survivor. For example, caregiver #13 shared that after her daughter finished treatment, her daughter no longer wanted to talk about cancer because she had “already been cured”. The caregiver recalled constantly saying, “Yes, my daughter, you are cured, but we have to continue talking. We just don’t know about the future”.

## 4. Discussion

Through qualitative interviews with caregivers, we yielded important insights regarding the reciprocal process of communication between caregivers and childhood cancer survivors, medical care teams, and families. Seven communication-related themes were identified, yet the most common themes that all participants reported were the importance and impact of discussing medical information and good communication experiences. Some caregivers discussed changes in how often they engaged in communication with others from the time of diagnosis to the end of treatment. Furthermore, only a few caregivers reported the need to engage in protective communication with family members, close friends, and, at times, the healthcare team. However, the key findings from the present study include the challenges of effective medical communications, non-medical discussions, and communication barriers for families who received care from a safety-net clinic in Los Angeles County.

We found that medical communication was often effective but nonetheless was inhibited by many barriers; this finding is consistent with the published literature. Even when medical communication of potential therapy side effects are clearly delivered, patients have reported that communication could be adversely affected by a perceived rush to treatment. In this situation, patients reported value in building a relationship with the treatment team and understanding options and next steps [21]. Among other factors, language barrier was a major barrier to successful communication amongst primarily Spanish-speaking caregivers; inconsistent interpreter use, telephone communications, and written communications were all reported barriers in a qualitative study among Spanish-speaking caregivers of children with medical complexity; and families reported often sharing a single cell phone or struggling to understand automated medication refill telephone services [22]. Physicians themselves could become a barrier to effective medical communication: issues identified include deterioration of communication skills over time, non-disclosure of key information, avoidance of difficult discussions, and discouragement of patient–provider collaboration and shared decision-making on the part of physicians [23]. Communication-impaired patients, in particular, are at greater risk of poor outcomes including disengagement from care and medical errors; utilizing a multidisciplinary team and improving physician training may help improve outcomes [24]. Of note, survivorship research has shown that concrete (written) care plans enhance overall understanding of treatments received and ongoing risks which lead to improved patient–provider communication [25].

Almost all the caregivers (13 out of 15) reported engaging in non-medical communication, which included discussions about social needs, return to normalcy, and future goals. The most prominent conversations included discussing their social needs with the healthcare team and expressing housing, transportation, finances, and employment challenges. These results suggest that childhood cancer survivors and caregivers are particularly vulnerable to experiencing social risk and social needs and, thus, should be routinely screened as part of routine care as it may impact their health and healthcare use [26,27,28,29,30]. For example, greater social risks can lead to a reduction or delay in seeking necessary healthcare. Furthermore, while social risks and social needs occur across the cancer continuum, they pose a disproportionate burden to underserved populations, thus likely contributing to disparities in cancer outcomes [31].

Finally, more than half of the caregivers (11 out of 15) indicated experiencing several barriers to communication with childhood cancer survivors, medical care teams, and family members. The most frequently disclosed factors were as follows: (1) the child’s age, (2) psychological hurdles and physical impediments to communication, and (3) lack of understanding and cultural and language differences. Combined, these findings underscore the critical role of building a strong interpersonal communication skill set for clinicians and empowering patients and caregivers with the necessary educational materials and toolsets to assist them in communicating their needs. Cancer survivors’ and caregivers’ quality of life is improved when they can adequately express their needs through medical and non-medical communication, and good communication may also have positive benefits (e.g., trust, accessibility, ability to express their concerns, or regular check-ins).

These findings complement and add to what is known about how communication can alter caregiver confidence and engagement in cancer therapy. It has been shown that caregivers learn about new diagnoses in a variety of manners, but reassuring provider communication that is consistent and well-paced in delivery enhances learning; in addition, it is important to note that the information communicated to patients and caregivers is not always the information they desired to know [32]. Caregivers reportedly feel more empowered to ask questions when clinicians use simple language, elicit concerns, encourage questions, and make ample available time for discussion; however, despite best efforts, caregivers report that they often feel too overwhelmed to ask questions. In the same study, caregivers also reported that they did not always know which team member to ask specific questions (nurse vs doctor vs other healthcare team member) [33]. Caregivers are more likely to feel acknowledged as a person of significance when they are well informed and receive honest communication; in contrast, a lack of communication, particularly at critical junctures such as the first discharge home or the cessation of therapy, resulted in a feeling of being an unwelcome guest, particularly if they feel the need to “nag” for information [34]. Effective communication clearly plays a large role in cementing the therapeutic alliance between the patient, caregiver, and provider.

Despite this study’s many strengths, there are limitations related to our recruitment and data collection procedures that should be considered. Related to our recruitment methods, since we recruited from a single clinic and used purposive sampling, our study participants were primarily mothers, and children predominantly had leukemia. Thus, these results may not be generalizable. As a result of the COVID-19 pandemic, our data collection was all carried out remotely by phone instead of face-to-face. However, notable strengths of this study encompass conducting interviews in both English and Spanish. Furthermore, employing the use of in-depth interviews allows for a comprehensive exploration of the perspective of understudied Hispanic/Latino caregivers.

## 5. Conclusions and Future Steps

In this qualitative study, thematic analysis of our semi-structured interviews found that overall, caregivers in our safety-net hospital caring for a primarily Hispanic/Latino immigrant community reported ongoing effectiveness and value in medical and non-medical communication. While the “first big talk” was particularly memorable, caregivers reported ongoing anxiety about uncertainty regarding cancer treatment and its impact on their child’s health. Caregivers participate in and respond to numerous non-medical communications, and the challenges of some of these conversations suggests the crucial role that the healthcare team can play in supporting the efforts of the caregivers regarding misinformation, social support, and the provision of hope. Further research is critical to understand understanding optimal ways to support Hispanic/Latino families in the myriad conversations encircling cancer care, and future steps for our team include exploring and understanding the hidden needs of underserved families regarding communication, relationships, and education along the journey to the end of therapy.

## Figures and Tables

**Table 1:** Self-reported participant demographics (*n* = 15).

Demographic Characteristics	*n* (%)
Caregiver	
Interview Language	
Spanish	10 (66.7%)
English	5 (33.3%)
Age in years, range	39 (range: 23–58)
Gender	
Male	1 (6.7%)
Female	14 (93.3%)
Marital status	
Single	2 (13.3%)
Married/Living with partner as married	10 (66.6%)
Divorced/Separated	3 (20%)
Highest education level	
Did not complete high school	9 (60%)
High school or equivalent	3 (20)
Some college/vocational training	3 (20%)
Household income	
<USD 20,000	5 (33.3%)
USD 20,000–USD 39,999	5 (33.3%)
USD 40,000–USD 59,999	3 (20%)
USD 60,000–USD 79,999	2 (13.3%)
Birthplace	
U.S. born	4 (27%)
Foreign-born	11 (73%)
Household size	Range: 5–9 persons
Type of health insurance	
Public	8 (53.3%)
Employment (private)	2 (13.3%)
None	5 (33.3%)
Childhood cancer survivor	
Cancer type	
Leukemia	10 (66.7%)
Hodgkin lymphoma	2 (13.3%)
Other ^1^	3 (20%)
Age at time of diagnosis	Range: 1–14 years
Current age	Range: 5–22 years
Gender	
Male	9 (60%)
Female	6 (40%)
Length of time since end of therapy	
<1 year	4 (26.7%)
1–2 years	2 (13.3%)
2+ years	9 (60%)

^1^ Other-type cancers consist of sarcoma, ovarian, and unknown (parent unable to recall type of cancer). Cancer type is self-reported by caregivers.

## Data Availability

The data presented in this study are available upon request from the corresponding author. Due to privacy restrictions, they are not publicly available.

## Supplementary Materials

The following supporting information can be downloaded at: https://www.mdpi.com/article/10.3390/healthcare12131307/s1. File S1: Qualitative interview instrument; File S2: Socio-demographic survey.
