# Supplementary material for: Healthcare Communication Experiences of Hispanic Caregivers of Childhood Cancer Survivors
Source: Healthcare (Basel). 2024 Jun 30;12(13):1307. doi: 10.3390/healthcare12131307 (PMC11241517; doi:10.3390/healthcare12131307)
Supplement: Supplementary file 1 [file healthcare-12-01307-s001.zip › healthcare-3063028-supplementary.pdf]

Supplemental File S1: Qualitative interview instrument

1. Can you share a bit about your experience as the primary caregiver of your child?
    a. Can you tell me about some of the challenges you have encountered providing care and support for your child as a result of his/her condition?
    b. Could you tell me about some of the positive things you have encountered providing care and support for your child as a result of his/her condition?
    c. What are some things that make it easier for you to take care of your child's health?
    d. What are some things that make it harder for you to take care of your child's health?

2. How have you been feeling since your child finished his/her cancer treatment?
    a. Can you describe how you think your mental health or well-being has been affected by your child's cancer experience?
    b. Are there other key sources of stress in your life that affect your ability to care for your child? If so, can you describe how this has impacted your caregiving?
    c. Have there been any changes to how you are feeling due to the current coronavirus pandemic?

3. Could you tell me how you think the cancer diagnosis and treatment have affected your child?
    a. Have you noticed any changes in your child's behavior since treatment?
    b. Have you noticed any changes given the current situation with the coronavirus pandemic?

4. How openly is your child's cancer diagnosis and treatment discussed between you and your child? What about in your household? And with doctors?
    a. Can you tell me about what type of cancer-related things you have talked about with your child, family, or doctors?
    b. Was there ever a time during your child's cancer treatment that you felt that you were unable to understand your child's medical provider?
    c. What about discussing any health issues about your child?

5. Can you please tell me a bit more about what it has been like for you to take care of your child during this current situation?
    a. What are your current main challenges as a result of the coronavirus pandemic?

6. We have talked about a lot of things today; is there anything else you would like to add?

Supplemental File S2: Socio-demographic survey

Section 1. Cancer survivors

1) How old is your child now? ______________ How old was your child when he/she was diagnosed? ______________
2) What is your relationship to the patient? ___________________
3) What was the name of the cancer or tumor your child was diagnosed with?
    - ☐ Any type of Leukemia (sometimes called ALL, AML, CLL, or CML)
    - ☐ Brain (or Central Nervous System)
    - ☐ Hodgkin's Lymphoma
    - ☐ NHL (non-Hodgkin's Lymphoma)
    - ☐ Bone tumor
    - ☐ Neuroblastoma
    - ☐ Other type: _________________________
    - ☐ Not sure of the type
4) Did your child's cancer treatment include chemotherapy?
    - ☐ Yes
    - ☐ No
    - ☐ Not sure
5) Did your child's cancer treatment include radiation therapy?
    - ☐ Yes
    - ☐ No
    - ☐ Not sure
6) Did your child's cancer treatment include surgery?
    - ☐ Yes
    - ☐ No
    - ☐ Not sure
7) About how long ago did your child finish his/her cancer treatment (of any type)?
    - ☐ Less than 1 year ago
    - ☐ 1-2 years ago
    - ☐ More than 2 years ago
    - ☐ Don't know
8) Does your child have any health problems as a result of his/her cancer and/or its treatment?
    - ☐ Yes
    - ☐ No
    - ☐ Not sure

IF YES:

7a) Please list problems/complications: ____________________________________

Section 2. Caregiver

1) What is your date of birth (dd/mm/yyyy)? __________________

2) In what country were you born?
   - ☐ United States
   - ☐ Other (please specify) __________________

3) How many years have you lived in this country? _____________ years

4) What is your gender?
   - ☐ Female
   - ☐ Male
   - ☐ Non-Binary
   - ☐ Prefer to self-describe: _____________
   - ☐ Prefer not to say

5) Which best describes your current marital status?
   - ☐ Single
   - ☐ Married
   - ☐ Living with a partner as married
   - ☐ Widowed
   - ☐ Divorced
   - ☐ Separated or no longer living as married

6) What is your household composition (including yourself)?__________________

7) Which category best describes the highest level of formal education you have completed?
   - ☐ Grade school (1-8 years)
   - ☐ Some high school (less than 12 years)
   - ☐ High school graduate or GED (12 years)
   - ☐ Some college, vocational or training school
   - ☐ Associate Degree (A.D. or A.A. degree)
   - ☐ College graduate (B.A. or B.S. degree)
   - ☐ Postgraduate education

8) How would you describe your employment status? **(Check all that apply)**
   - ☐ Working full-time
   - ☐ Working part-time
   - ☐ Stay-at-home parent
   - ☐ Student

- ☐ Unemployed
- ☐ Other: ___________________________________

9) Which of the following income groups represent your total family income for the past 12 months?
   - ☐ Less than $10,000
   - ☐ $10,000-19,999
   - ☐ $20,000 to less than $29,999
   - ☐ $30,000 to less than $39,999
   - ☐ $40,000 to less than $49,999
   - ☐ $50,000 to less than $59,999
   - ☐ $60,000 or more
   - ☐ Decline to state

10) What type of health insurance or health care coverage do you currently have?
   - ☐ None
   - ☐ Public (Such as Medicaid)
   - ☐ Private health insurance or HMO (such as Blue Cross or Kaiser)
   - ☐ Other: ________________________
   - ☐ Don't know
